# Subjective and Autonomic Arousal toward Emotional Stimuli in Preadolescents with Externalizing Problems and the Role of Explicit and Implicit Emotion Regulation

**DOI:** 10.3390/brainsci14010084

**Published:** 2024-01-16

**Authors:** Maria Panteli, Thekla Constantinou, Andry Vrachimi-Souroulla, Kostas Fanti, Georgia Panayiotou

**Affiliations:** 1 Department of Psychology, University of Cyprus, Nicosia 1678, Cyprus; 2 Center for Applied Neuroscience, University of Cyprus, Nicosia 1678, Cyprus

**Keywords:** externalizing problems, arousal, emotion regulation, conscious emotion regulation, emotional awareness, heart rate variability

## Abstract

Children and adolescents with externalizing problems show physiological hypo-reactivity toward affective stimuli, which may relate to their disruptive, antisocial, and thrill-seeking behaviors. This study examines differences in explicit and implicit emotion regulation between preadolescents with and without externalizing problems as well as the role of emotion regulation in subjective and autonomic responses to emotional stimuli. Preadolescents showing self- and other-reported externalizing psychopathology, and a control sample, without such difficulties, participated in a passive affective picture-viewing task with neutral, fearful, joyful, and sad images, while their heart rate and heart rate variability were measured. Participants also reported on their emotion regulation difficulties using the Difficulties in Emotion Regulation Scale. Compared to controls, youths scoring high on externalizing problems (1) reported greater emotion regulation difficulties, especially a lack of emotional clarity and difficulty in controlling impulsive actions, (2) showed higher resting heart rate variability and a lower resting heart rate, suggestive of higher emotion/autonomic regulation ability, and (3) showed both subjective and physiological hypo-arousal to emotional pictures. Heart rate variability and, to a lesser degree difficulties in emotional clarity, modulated the effects of emotional pictures on subjective and physiological arousal. Findings suggest that interventions to improve emotion regulation and awareness may help to prevent externalizing problems.

## 1. Introduction

Emotion regulation plays a critical role in many forms of psychopathology [1,2] and predicts treatment outcomes and clinical severity [3,4]. However, limited evidence of its role in childhood disorders exists, especially with the use of both objective and subjective measures. This is a critical gap as children may be limited in their ability to report on their internal experiences. Focusing on multiple levels of analysis of emotion regulation processes, i.e., both subjective and physiological, is also consistent with the Research Domain Criteria framework [5,6] and allows for the identification of biological markers that can predict psychopathological processes. The present study aims to explicate the role of emotion regulation, assessed with self-report and psychophysiological methods, in the emotional responses of youths with externalizing problems [7,8,9]. Typically, their responses are consistent with both subjective and physiological under-arousal. This pattern is considered a downward developmental extension of the restricted reactivity to negative stimuli observed in antisocial adults [10,11], which has been implicated in the etiology and maintenance of their difficulties. In this study, we evaluate the hypothesis that observed hypo-reactivity among youths is related to atypical explicit or implicit emotion regulation processes.

Substantial evidence supports the claim that externalizing symptoms in childhood, especially conduct problems, are associated with deficits in the arousal system. This assertion has been documented by findings of decreased heart rate (HR) and/or skin conductance responses to emotional stimuli [12], such as fearful or aversive stimuli [7], as well as contexts involving orienting or social stressors [13,14,15,16,17]. Resting HR is also reduced in these samples, and in fact, a low resting heart rate in the mildly stressful context of a psychophysiology lab has been considered a reliable characteristic of antisocial youth [18,19]. 

Such findings, despite some inconsistencies with regard to the specific clusters of symptoms that predict them and the emotional contexts in which they present, are in accord with both the hypo-arousal and the fearlessness hypotheses of childhood conduct problems [20]. These hypotheses posit that decreased autonomic responses, either specifically in fear and distress situations or in aversive emotional contexts more broadly [8,9] could result in a failure to learn from the consequences of behavior, a necessary process for shaping social conscience. At the same time, restricted arousal to emotional situations can lead to unpleasant feelings of boredom and, in turn, sensation- and thrill-seeking [21] behaviors. We suggest that emotion regulation may play an important role in these effects.

Emotion regulation at a conscious/explicit level includes the implementation of processes of emotional awareness and understanding, acceptance of affective states, control of impulsive actions, and the initiation of behaviors to pursue desired goals. It also entails the ability to use self-regulatory processes in a flexible manner in accordance with environmental demands and personal values [22]. Explicit emotion regulation processes are typically assessed using self-report tools, like the Difficulties in Emotion Regulation Scale (DERS) [4,22,23]. 

At the implicit level, various physiological and homeostatic processes take place dynamically throughout the stages of emotion processing [24]. Heart rate variability (HRV) can be used to index automatic flexibility and regulation, i.e., the continuous interplay between sympathetic and parasympathetic nervous system activity. It has been considered as an objective measure of regulated emotional responding and trait-like emotion regulation ability [25]. At the implicit level, adaptive regulation would entail the ability to show appropriate arousal in intense or relevant emotional contexts but downregulate responses when these are extreme or when the stimulus is no longer present or relevant (e.g., during rest). 

Prior findings on explicit and implicit emotion regulation in individuals with externalizing problems are limited. With regards to the DERS, both internalizing and externalizing problems have been related to emotion regulation difficulties [26]. Specific difficulties were identified in strategies involving emotional awareness, clarity, and the use of impulsive actions, sometimes found to specifically characterize youths with externalizing difficulties (e.g., [4,27]). Lower overall HRV was observed in some studies of children with externalizing problems relative to healthy controls, as indexed by various HRV parameters. These parameters include respiratory sinus arrhythmia (RSA), the root mean square of successive differences (i.e., RMSSD), the proportion of NN50 divided by the total number of NN/R-R intervals (i.e., pNN50), and the standard deviation of NN intervals (i.e., SDNN) [28,29]. However, others showed increased RSA in this population compared to controls [30] and a positive correlation between oppositional defiant disorder symptoms and the High Frequency (HF) domain of HRV [31], indicating good parasympathetic control and emotion regulation. Still, other findings show no differences in sympathovagal balance between youths with externalizing problems and healthy controls [32]. These contradictory findings point to the need for further studies and more precise definitions of sample characteristics and symptomatology. For example, conceptualizing children with externalizing problems as emotionally unresponsive may yield hypotheses claiming that they would show emotional over-regulation (i.e., high HRV) to reflect their restricted emotionality, while conceptualizing them as impulsive and dysregulated would suggest reduced HRV. However, because of the acknowledged heterogeneity of this population [33], with regards to the presence of comorbid internalizing symptoms, other pathogenic traits, and characteristics, further clarification of this association is required. 

## 2. Current Study

### Objectives and Hypotheses

The present study aims to address two main questions: First, to test whether preadolescents with and without externalizing problems differ in emotion regulation ability, as measured with self-report (DERS total and sub-scale scores) and resting HRV. In particular, SDNN and RMSSD will be assessed, as they are considered accurate indicators of overall HRV and parasympathetically mediated HRV, respectively [25,34]. 

Based on prior evidence on explicit emotion regulation, we expected self-reported difficulties in regulating emotions. Additionally, given the mixed findings, no a priori directional hypotheses could be posited regarding HRV. 

Second, we aimed to examine if differences in emotion regulation ability could in part account for the expected atypical subjective and autonomic emotional responses. These responses are indicative of reduced arousal, previously found among youth with externalizing problems. Specifically, we aimed first to replicate group differences in emotional under-reactivity and subjective and physiological reactivity in response to negative emotional contexts. We hypothesized that the group differences would no longer be significant when the effect of emotion regulation is removed. This would indicate that emotion regulation as assessed by the DERS and/or HRV modulates arousal reactivity differences. 

Documenting that emotion regulation is implicated in the emotional deficits of youths with externalizing problems has important implications for psychological interventions with this population. Emotion regulation skills are malleable to treatment, for example, through emotion regulation skills training. Therefore, improving these skills can represent an impactful pathway to prevent the ramifications of externalizing problems on children’s well-being and future adaptation. 

## 3. Method

### 3.1. Participants

For this study, we used data from a larger project assessing the cognitive and emotional profiles of children with behavioral problems. This project was approved by the Cyprus National Bioethics Committee. Other aspects of the project were published in Souroulla et al. [7]. Eighty-seven Greek-Cypriot children (45 males, 42 females) between the ages of 10 and 12 (*M_age_* = 11.20, *SD* = 0.65 years) were recruited from randomly selected urban and suburban elementary schools of the Nicosia district. 

Specifically, from a pool of 125 elementary schools in the district (based on the Ministry of Education and Culture records), 18 schools were chosen randomly. Out of the 18 schools, 17 agreed to participate, with only one declining due to scheduling conflicts. Although schools were selected randomly, participants were selected to fit into either a clinical group or a control group. Specifically, the involved schools nominated children showing behavioral problems and invited parents of both the nominated children and the rest of the children in the same classes to provide consent for participation in the study. After receiving written consent from their parents, all children were assessed with structured clinical interviews for clinically significant externalizing symptoms of ADHD, conduct disorder, or oppositional defiant disorder. 

**Figure 1:**
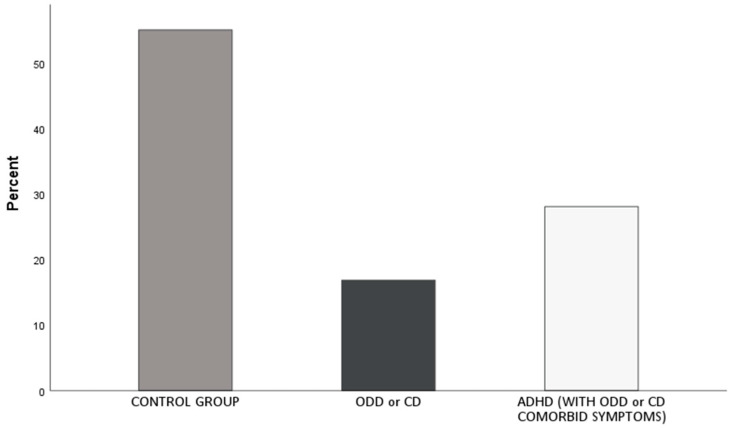
Composition of the experimental sample after the selection process. The clinical group is composed of the participants categorized as ODD or CD and ADHD, as shown in the last two columns.

The clinical interviews with the children were conducted with the Mini-International Neuropsychiatric Interview for Children and Adolescents (MINI-KID). Participants meeting the diagnostic criteria on the structured interview, for at least one externalizing disorder, i.e., ADHD, conduct disorder, or oppositional defiant disorder were assigned to the clinical group (*n* = 39; *M_age_* = 11.44). Children with externalizing symptomatology were placed into a single group due to the high degree of comorbid symptoms of the externalizing disorders examined. Alternatively, those who did not meet the diagnostic criteria for any of these conditions were assigned to the control group (*n* = 48; *M_age_* = 11.03, see Figure 1). 

Recruited preadolescents had normal intelligence and did not exhibit any sensory, psychotic, or pervasive developmental disorders or receive psychotropic medications. Six children completed the questionnaires but declined to take part in the experiment. Seventy-five children completed the experiment, while the rest terminated participation before completion due to other scheduled activities. After cases were removed listwise for technical issues and noisy signals, HR analyses included 74 participants and HRV 71 participants. 

### 3.2. Experimental Procedure

During the experiment, children completed a passive picture-viewing task during which they were exposed to four picture types: neutral, joyful, fearful, and sad. This experimental paradigm follows similar studies with youth and children [35,36]. 

Upon arrival at the laboratory, participants and their guardians were fully informed about the procedure and provided written consent. The participants sat in a reclining chair and were instructed to clear their minds of any thoughts and feelings and try to relax. The attachment of electrodes and instructions were followed by a five-minute period to stabilize physiological signals and familiarize children with the equipment. A seven-minute relaxation period followed, during which resting heart rate was recorded. A series of cognitive tasks irrelevant to this study followed before the start of the reported experiment.

For the passive viewing task, the participants were asked to focus their attention on the pictures. The pictures were presented in one of three pseudorandom orders on a 45-inch TV monitor and lasted for six seconds each. All stimuli were presented using E-Prime 2.0 software in a random order, twice. Pictures were separated by a random inter-trial interval of 7, 12, or 18 s to reduce onset predictability. 

After the end of the viewing task, pictures were presented again, and the preadolescents rated their experienced arousal, dominance, and valence during each picture using a five-point scale and paper-and-pencil modification of the Self-Assessment Manikin (SAM) [37]. Finally, participants were debriefed and received a gift of 15 Euros and a toy.

### 3.3. Material

**Emotional Pictures.** Pictures were selected from locally normed stimuli from the International Affective Picture System (IAPS) [38]. The chosen pictures represent the four quadrants of affective space, i.e., positive and negative with high arousal ratings (joy, fear), and neutral/mildly positive and negative with low arousal ratings (neutral, sad; see Souroulla et al. [7]). 

### 3.4. Apparatus

BIOPAC MP150 for Windows and Acq3.9 data acquisition software (version number: 3.9.0.17) (Biopac Systems Inc., Santa Barbara, CA, USA) were used to collect psychophysiological signals. HR was assessed with Lead I EKG in beats per minute (BPM) using Ag/AgCl disposable electrodes placed on the forearms (filtered by a BIOPAC ECG100C bioamplifier). 

The inter-beat intervals for HRV analyses were obtained from EKG and were visually inspected for artifacts; IBIs were written in a text file, and their variability was estimated by the ARTiiFACT program [39] with cubic spline interpolation correction. For this study, we used the SDNN and RMSSD HRV indices. 

During data reduction, mean raw BPM were offline processed. Outliers above ±2.5 SD within each person for each measurement were removed, and the means were computed for each picture trial and ITI and, finally, for each condition (neutral, fear, joy, sad). 

### 3.5. Measures

**Mental Health.** The Mini-International Neuropsychiatric Interview for Children and Adolescents (MINI-KID child version) [40] is a short, validated, and structured diagnostic tool based on DSM-IV and ICD-10 [40] for 30 diagnostic categories (e.g., depressive disorders, anxiety disorders, ADHD, and disruptive disorders). It was modified for youths but follows the same question format as the original MINI neuropsychiatric interview [41,42]. It is considered suitable for children between 4 and 17 years of age and is a reliable diagnostic tool for use in general and clinical samples [43]. For example, the Major Depression section, starts with the question “In the past two weeks when you felt depressed/grouchy/uninterested were you less hungry or more hungry most days? Did you lose or gain weight without trying?…”. All items are answered yes or no, and the corresponding questions are asked about possible previous episodes of the disorder. The structured interview was conducted with the child and scored by Clinical Psychology Ph.D. students under the supervision of a licensed clinical psychologist. 

**Emotion Regulation Difficulties.** The Self-reported Difficulties in Emotion Regulation Scale (DERS) [22] was used to measure perceived emotion regulation difficulties in preadolescents. This scale, translated into Greek, includes 36 items divided into six subscales: lack of emotional clarity (5 items; e.g., “I have no idea how I am feeling”), lack of emotional awareness (6 items; e.g., “I am attentive to my feelings”-reverse), difficulties to control impulsive actions (6 items; e.g., “When I’m upset, I feel out of control“), difficulties to engage in goal-directed actions (5 items; e.g., “When I’m upset, I have difficulty concentrating”), non-acceptance of emotions (6 items; “When I’m upset, I become angry with myself for feeling that way”), limited access to emotion regulation strategies (8 items; e.g., “When I’m upset, I believe that I’ll end up feeling very depressed”). The items are rated on a 5-point Likert scale ranging from 1 (almost never) to 5 (almost always). The DERS has good psychometric properties; in the original standardization study, Cronbach’s alpha for the total scale was 0.93 and, in the current study, it was 0.88. Internal consistency for each subscale ranged within acceptable limits (limited access to emotion regulation strategies a = 0.75; non-acceptance of emotions a = 0.78; difficulties to engage in goal-directed actions a = 0.79; difficulties to control impulsive actions a = 0.79; difficulties to control impulsive actions a = 0.79; lack of emotional awareness a = 0.73; lack of emotional clarity a = 0.52). The somewhat lower reliability shown by the lack of emotional clarity subfactor has been found by others as well (e.g., a = 0.54 [44]). Lower reliabilities are to be expected for scales composed of a few items [45], with 0.5 considered low but acceptable [46]. 

**Subjective Emotion.** Ratings for arousal, valence, and dominance during each picture were obtained using a paper-and-pencil version of SAM [37] and a 5-point rating scale; for arousal, examined in the present study, responses ranged from 1 (high arousal) to 5 (low arousal), which were reverse scored for easier interpretation at the stage of analysis. 

## 4. Statistical Analysis

SPSS 27 for Windows was used for analyses. We examined with separate one-way MANCOVAs the differences on the DERS and DERS subscales and, separately, baseline HR and HRV between preadolescents with and without externalizing psychopathologies, controlling for age as a covariate. Separate repeated measures ANOVAs (rANOVA) were conducted for HR and subjective arousal during picture viewing. The picture type was entered as an interaction of 2 valence levels (positive and negative) * 2 arousal levels (high and low), to contrast more positive emotions with more negative emotions and high arousal with low arousal ones. The group variable (with two levels clinical and control) was the between-subject factor. The rANOVAs were next repeated as rANCOVAs, entering DERS or baseline HRV as covariates in separate analyses, when significant main effects of group, or interactive effects of group were observed. 

## 5. Results

### 5.1. Group Differences in Emotion Regulation 

Based on MANCOVA controlling for age, we found a significant difference between the externalizing and the control group in the total DERS score and specific DERS subscales. A significant effect of group was found based on the group, *F* (6, 80) = 3.70, *p* = 0.003; Wilk’s lambda = 0.78, partial eta squared = 0.22. Namely, differences in the control of impulsive actions and lack of emotional clarity were found (Table 1), with the externalizing group reporting greater difficulties than controls, as expected. The remaining scales resulted in non-significant differences. 

The corresponding MANCOVA for baseline autonomic indices (heart rate and heart rate variability) also resulted in a significant effect of the group, *F* (3, 66) = 4.80, *p* = 0.004; Wilk’s lambda = 0.82, partial eta squared = 0.18. The two groups differed in HRV, with the difference being significant for SDNN, such that participants with externalizing problems had higher resting HRV than controls (Table 1). Group differences in RMMSD fell short of significance, but the group means were in the same direction as for the SDNN (i.e., better autonomic regulation for externalizing participants). The resting HR difference was also significant, with lower HR for the externalizing group, as expected.

### 5.2. Affective Arousal Responses to Emotional Contexts

**Subjective Arousal.** The rANOVA for subjective arousal showed a significant main effect of the emotional arousal of the pictures, *F* (1, 79) = 124.58, *p* < 0.001, η_p_^2^ = 0.61, no significant main effect of the emotional valence, and a significant emotional valence x emotional arousal interaction, *F* (1, 79) = 30.285, *p* < 0.001, η_p_^2^ = 0.274, suggesting that high-arousal emotional contexts (joy *M* = 3.73, *SD* = 1.26; fear *M* = 3.38, *SD* = 1.04) resulted in significantly higher arousal ratings than low-arousal emotions (neutral *M* = 2.05, *SD* = 1.06, sad *M* = 2.95, *SD* = 1.10) and that, for negative emotions, fear resulted in higher ratings than sadness and, for the more positive emotions, joy resulted in higher ratings than neutral. There was no main group effect and no significant emotional arousal × group interaction. Instead, a significant emotion valence × group interaction, *F* (1, 79) = 5.24, *p* = 0.03, η_p_^2^ = 0.062, was identified, showing that groups responded differently to positive vs. negative emotional conditions. 

**Figure 2:**
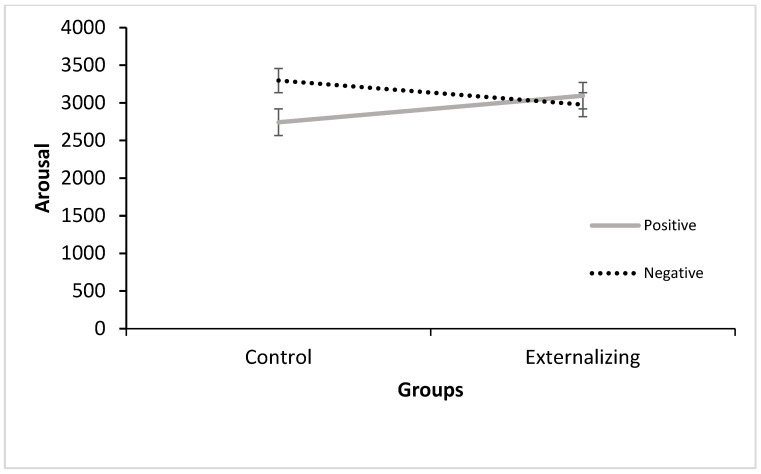
Subjective Arousal Ratings for positive and negative pictures (Valence) for each group (Control *n* = 47; Externalizing *n* = 34).

Decomposing this interaction through post hoc contrasts showed that the control group reported higher subjective arousal during negative pictures (Fear *M* = 3.50, *SD* = 0.95; Sad *M* = 3.08, *SD* = 1.04) compared to the externalizing group (Fear *M* = 3.19, *SD* = 1.55; Sad *M* = 2.75, *SD* = 1.15), *F* (1, 79) = 3.83, *p* = 0.05, η_p_^2^ = 0.046, whereas the opposite pattern was observed for positive pictures, with the externalizing group reporting more arousal (Joy *M* = 3.99, *SD* = 1.21; Neutral *M* = 2.19, *SD* = 1.07) than the control group (Joy *M* = 3.53, *SD* = 1.27; Neutral *M* = 1.94, *SD* = 1.05). The latter contrast, however, did not reach significance, *F* (1, 79) = 2.680, *p* = 0.106, η_p_^2^ = 0.11 (Figure 2).

**Figure 3:**
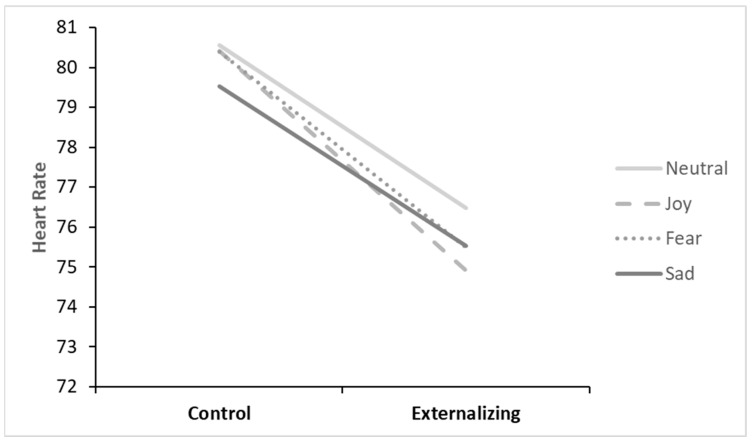
Heart rate responses across experimental conditions and groups (Control *n* = 43, Externalizing *n* = 33).

**Physiological Arousal.** The rANOVA for HR during picture viewing showed a significant group effect, where the externalizing group had significantly lower HR across emotional conditions than the control group, *F* (1, 74) = 3.93, *p* = 0.05, η_p_^2^ = 0.05 (Figure 3). An emotional valence x emotional arousal interaction, *F* (1, 74) = 15.01, *p* < 0.001, η_p_^2^ = 0.169, indicated that, across groups, HR differed for different emotional contexts. Specifically, post hoc comparisons showed that within high-arousal emotions, fear (*M* = 77.93, *SE* = 1.40) resulted in higher HR than joy (*M* = 77.02, *SE* = 1.42), *F* (1, 74) = 5.25, *p* = 0.025, η_p_^2^ = 0.066, and neutral (*M* = 77.98, *SE* = 1.41) resulted in higher HR than sadness (*M* = 76.98, *SE* = 1.37), which had the lowest overall HR response, *F* (1, 74) = 8.30, *p* = 0.005, η_p_^2^ = 0.101. 

### 5.3. Role of Emotion Regulation on Group Differences in Arousal

**Role of Explicit Emotion Regulation in Subjective Arousal:** To examine whether emotion regulation is implicated in group effects on subjective ratings (i.e., the valence x group interaction), DERS parameters on which the groups scored differently were entered in separate rANCOVAs (otherwise identical to the rANOVAs above) as covariates. When the DERS total score was entered as the covariate, the emotional valence x group interaction remained significant, *F* (1, 77) = 4.658, *p* = 0.034, η_p_^2^ = 0.057, indicating that conscious/explicit emotion regulation does not affect differential group subjective responses to positive and negative emotions. However, the main emotional arousal effect, *F* (1, 77) = 1.522, *p* = 0.22, η_p_^2^ = 0.019, and the emotional arousal x emotional valence interaction, *F* (1, 77) = 0.001, *p* = 0.98, were no longer significant, indicating that emotion regulation ability is related to subjective emotional responses irrespective of groups. 

Next, DERS clarity and DERS impulsive actions were entered as covariates in separate analyses. When an impulsive action was entered, both the main and interactive effects remained significant, suggesting that this aspect of conscious emotion regulation is not related to subjective arousal responses. The same held for clarity as the covariate, with the exception that the emotional valence x emotional arousal interaction stopped being significant. This suggests that emotion regulation through emotional clarity may be related to more fine-grained subjective arousal responses, across participant groups.

**Role of Implicit Emotion Regulation in Subjective Arousal:** Similar rANCOVAs were conducted with HRV as the covariate, specifically SDNN, the parameter on which the groups differed significantly. Although all other effects remained, the emotional valence x group interaction now fell short of significance, (*F* (1, 69) = 3.207, *p* = 0.078, η_p_^2^ = 0.044). This suggests that group differences in the ability to differentiate among emotions of different valences in their subjective responses, and specifically the difficulty of the externalizing group, compared to the control group in this regard, are affected by the regulation of autonomic responses.

### 5.4. Role of Emotion Regulation in Physiological Arousal

Repeated measures of ANCOVAs were used to examine if emotion regulation modulated the significant main group effect on HR, entering DERS parameters and SDNN as covariates one at a time. In the case of the total DERS and use of impulsive action, the group effect decreased to marginal (*p* = 0.08 and 0.07, respectively), while in the case of emotional clarity and SDNN, the group effect was no longer significant (*p* = 0.15 and 0.46, respectively). The findings suggest that both explicit (especially regulation through emotional clarity) and implicit (SDNN) emotion regulation are implicated in group differences in autonomic responses to emotions. 

## 6. Discussion

The present study contributes new findings that expand our understanding of the role of emotion regulation processes in childhood externalizing psychopathology. The study’s significance lies in our multi-level approach, which is consistent with contemporary theoretical frameworks that propose the examination of mechanisms of psychopathology at both subjective and biological levels of analysis (e.g., [10]). The value of this approach is underscored by the findings themselves, which demonstrated that conscious/explicit and physiological/implicit emotion regulation processes do not necessarily covary; a multi-level analysis may be needed to gain a fuller understanding of the role of emotion regulation in psychopathology. Our findings support the presence of emotion regulation differences between typically developing children and those with externalizing problems. They also suggest a significant role of this difference in the hypo-aroused emotional responses seen in externalizing pathology.

Regarding our first research question, we found greater overall difficulties in the conscious use of emotion regulation strategies reported by preadolescents with externalizing problems and more specifically in the use of impulsive behaviors and lack of emotional clarity. These findings are similar to previous reports of difficulties among youths with externalizing behaviors in overall DERS scores [47], in the use of impulsive actions, in emotional awareness, and in some studies in emotional clarity, compared to controls [4,27,48]. Although here we found differences in emotional clarity rather than awareness, these are related processes. Awareness is necessary for clear, granulated perceptions of one’s emotional experience. Youths with externalizing problems may lack full knowledge of subjective feelings and the ability to use this to cope with challenges. 

Also, consistent with a limited number of previous findings, the externalizing group showed greater trait-like resting autonomic regulation, based on SDNN, an overall marker of HRV, relative to the control group. This pattern suggests the ability to regulate autonomic emotional responses. The decoupling between poor emotion regulation on subjective measures and good emotion regulation on objective, physiological indices among the externalizing group is a noteworthy finding. It corresponds in part with previous evidence of, on the one hand, high scores on subjective/observer-reported emotional dysregulation in this population [47], and on the other hand, higher HRV [28,30]. A previous investigation [48] that examined the correlation between the two assessment methods among children with internalizing and externalizing problems observed that they follow the same direction (i.e., higher RSA measured at three time points was related to fewer DERS difficulties at time 3). However, notably, among children who scored high on DERS at time 3, RSA did not seem to change significantly across the three time points, in contrast to children with low emotion regulation difficulties, suggesting that the covariation between DERS and RSA is stronger among those without emotion regulation problems. This pattern is similar to our finding of a decoupling between conscious and implicit regulation processes. 

In our own sample, the divergent findings for HRV and DERS indices of emotion regulation may suggest that, in their own perception, preadolescents with externalizing problems have difficulties in using emotion regulation strategies when needed. However, at the same time, their own autonomic system effectively suppresses extreme emotional reactions. Good autonomic regulation among youths with externalizing difficulties may reduce the intensity of physiological emotional cues, which may in turn prohibit conscious engagement in appropriate emotion regulation strategies. 

Downregulation of physiological arousal in the externalizing group was evident not only in HRV parameters but also in their overall HR both during picture viewing and during rest, consistent with the plethora of evidence for autonomic hypo-reactivity in this population. According to prominent theories [49], awareness of emotions, for which interoceptive cues are a necessary component, is required for the deployment of adaptive emotion regulation responses. This ability is apparently reduced among these youth, especially considering their difficulties in emotional clarity. Interestingly, despite their arousal being well-regulated, the externalizing group’s reports of difficulties with the DERS suggest that they believe they should be able to control their emotions even more, indicating perhaps that emotions, especially the negative ones that typically trigger emotion regulation [50,51], are perceived as unwanted.

The non-significant differentiation in subjective arousal between positive and negative emotions observed among the externalizing group, but not among the control group, is an additional noteworthy finding, which may be implicated in the ability of the former to recognize threatening, sad, or otherwise challenging situations [52]. This difficulty may result in more risk-taking, aggressive, or unempathetic behaviors. This is consistent with fearfulness and hypo-arousal hypotheses that postulate deficiencies in learning from the consequences of behavior in samples with externalizing problems, due to the absence of aversive affective cues, for example, after behavior that causes fear or sadness in others. The absence of internal cues to trigger conscious emotion regulation may deprive these children of the opportunity to practice coping and emotion regulation skills, in order to develop adaptive emotional and interpersonal responses.

The current study also contributes new findings on the role of emotion regulation (explicit and implicit) in the physiological affective hypo-reactivity of those with externalizing problems based on ANCOVA results. Specifically, some of the differences between the two groups in subjective arousal responses were no longer significant when emotion regulation parameters were entered as covariates, which suggests that emotion regulation explains some of the variance in the observed group differences. Alternative statistical explanations of the effect include that emotion regulation mediates group differences in emotional arousal, or that it relates to both group status and arousal effects, acting as a confounding variable. In any case, the results show that emotion regulation contributes to variance that is significantly implicated in group differences in arousal, which should be carefully considered.

Specifically, findings suggest that SDNN but not explicit emotion regulation affects subjective response differences between the two groups. Instead, strategies related to emotional clarity seemed to be involved in subjective arousal responses irrespective of the group. Both SDNN and subjective emotional clarity on the DERS modulated HR responses to emotional pictures, consistent with findings that emotion regulation enables individuals to keep internal levels of arousal in a performance-optimizing range [53]. 

**Clinical Implications:** These findings have implications for clinical interventions. Although directly changing HRV processes may not be an easy goal of psychological therapies, training youths who have reported emotion regulation difficulties in increased emotional clarity, awareness, and granulation may be beneficial to their emotional health. It may facilitate them to utilize information provided by their own emotions, including arousal-related physiological cues, toward more adaptive behavior, in the direction of reducing impulsive, sensation-seeking actions, and improving interpersonal relationships. Findings are in line with arguments that emotion regulation is a transdiagnostic factor in the development of behavioral problems [54]. Interventions focused on emotion regulation such as Dialectical Behavioral Therapy have already shown promise for adolescents with behavioral problems, including evidence of reductions in impulsive actions, use of maladaptive emotion regulation strategies, externalizing behaviors, and anger management [55,56]. 

Socio-emotional learning programs, which focus on the development of emotion regulation skills, implemented more universally may also show promise in preventing externalizing problems before they appear [57]. Future interventions can target specific skill deficits, e.g., emotional awareness and clarity and the reduction of impulsive actions, and test their effectiveness, by showing change in both explicit and implicit emotion regulation measures.

**Study Limitations:** The current study comes with some limitations. The sample size is relatively small, although considering the statistical power of similar studies suggests that the number of participants may have been adequate (e.g., [58]). However, our findings need to be replicated in larger samples and groups with specific externalizing symptoms and additional characteristics that can address the impact of the heterogeneity of youths with these types of difficulties on current findings. Specifically, the externalizing sample in this group mostly showed comorbidity for more than one externalizing disorder and was nominated by schools as having apparent difficulties, which suggests that the severity was probably high. Other populations with specific single diagnoses that exclude any comorbidities may show different results. Also, given that the DERS items focused mainly on difficulties in dealing with unpleasant affective states, further studies using measures of explicit regulation of pleasant emotions are needed to provide a more comprehensive picture of the emotion regulation difficulties of youths with externalizing psychopathology. Relatedly, although within acceptable limits, the reliability of the clarity scale was rather low, and therefore, results pertaining to this strategy should be seen with some caution. Lastly, following well-established passive picture-viewing paradigms, we only exposed participants to the stimuli for 6 s each, precluding the analysis of HRV during emotional processing, which would have been ideal for understanding dynamic implicit regulation processes. In defense of our design, DERS was also completed before the experiment, allowing for comparative timeframes of implicit and explicit emotion regulation processes.

## 7. Conclusions

In summary, this is the first study to our knowledge that attempts to explore the role of both subjective and biological emotion regulation abilities in the atypical emotional responses of youths with externalizing psychopathology. Our findings point to a decoupling between explicit and implicit emotion regulation in this population, which should be taken into account when assessing their emotional difficulties. The results suggest the potential utility of interventions targeting the development of emotion regulation skills, especially skills to control impulsive actions and enhance emotional clarity. These are expected to enhance the ability and perceived competency of youths with externalizing problems to cope with aversive emotional situations. This may in turn translate into less disruptive and aggressive behaviors and the ability to use emotional information to cope with life challenges and adaptively pursue personal goals. 

## Figures and Tables

**Table 1:** Means (M), Standard Deviations (SD), and MANCOVAs for DERS, resting HR, and HRV.

Measure	Externalizing Group (*n* = 40)		Control Group (*n* = 48)		*F* (df)	*p*
	*M*	*SD*	*M*	*SD*		
DERS Total score	88.28	18.18	76.29	20.42	10.68 (1, 85)	*p* = 0.002
DERS subscales						
Control of impulsive actions	15.58	5.68	11.85	5.28	9.66 (1, 85)	*p* = 0.003
Lack of emotional clarity	11.10	3.49	8.96	3.09	13.31 (1, 85)	*p <* 0.001
Restricted access to emotion regulation strategies	18.30	5.45	15.90	6.13	6.37 (1, 85)	*p* = 0.013
Non-acceptance of emotions	12.30	5.37	11.23	4.77	1.96 (1, 85)	*p* = 0.16
Lack of awareness	17.20	6.11	15.69	3.34	1.96 (1, 85)	*p* = 0.18
Difficulty to engage in goal-directed behaviors	13.80	4.61	12.67	5.85	1.12 (1, 85)	*p* = 0.29
SDNN	74.44 (*n* = 31)	29.25	60.51 (*n* = 41)	24.23	3.31 (1, 63)	*p* = 0.074
RMSSD	68.15 (*n* = 31)	30.04	58.75 (*n* = 41)	31.23	0.701 (1, 63)	*p* = 0.40
Resting HR	75.13 (*n* = 28)	11.01	82.01 (*n* = 36)	12.17	4.349 (1,63)	*p* = 0.041

Note. DERS = Difficulties in Emotion Regulation Scale (Gratz and Roemer, 2004 [22]). Higher scores on DERS and DERS subscales indicate greater difficulties. SDNN = Standard deviation of N-N intervals. RMSSD = Root mean square of successive RR interval differences. Resting HR = Resting Heart Rate. Differences in sample numbers in physiological analyses are due to the deletion of cases listwise for poor signal measurement.

## Data Availability

The data presented in this study are available on request from the corresponding author. The data are not publicly available due to ethical restrictions because no consent for open data sharing was secured from participants.
